# Apicotomy: a root apical fracture for surgical treatment of impacted upper canines

**DOI:** 10.1186/1746-160X-3-33

**Published:** 2007-09-06

**Authors:** Edela Puricelli

**Affiliations:** 1 School of Dentistry, Federal University of Rio Grande do Sul, Porto Alegre, RS, Brazil

## Abstract

Impacted canines, due to systemic or local factors, represent a frequent problem in most populations. Surgical intervention usually involves exposure for spontaneous eruption, exposure for orthodontic traction or extraction. The author presents the apicotomy technique, which has been successfully used during the past twenty years for conservative intervention in cases of impacted upper canines with dilaceration or apical root-ankylosis. This original method involves surgical fracture of the root apex, followed by orthodontic traction of the corono-radicular region.

## Background

Canines guide the teeth into proper bite, and have therefore specific functions in chewing and in excursive movements of the mandible. According to Fiedler and Alling in 1968 [1] and Mead and Monsen in 1965 [2], canines present proprioceptive and reflexive fibres which protect and stabilize occlusion. Due to their position, they provide an aesthetic and harmonious transition between the anterior and posterior segments of the dental arch. Canines have the longest roots and are the most resistant teeth [3,4] and thus are often displaced or impacted [5]. The prevalence of maxillary canine impaction seems to be related to the ethnic origin [6]. The lowest frequency (0.27%) is seen among Japanese individuals [7], while the highest (1.8%) is observed in Iceland [8]. Impacted canines occur more frequently in females than males, with a proportion of 2.5:1 [9].

Maxillary canines travel a long, tortuous path before they erupt, and the long axis may adopt an inclined or horizontal position related to the occlusal plane. Impaction might occur due to general or local factors. The etiopathological investigation of impaction may reveal the existence of systemic diseases such as cleidocranial dysplasia, or Gardner and Gorlin-Goltz syndromes. However, many local problems may be involved, particularly those related to alterations in bone or dental structures and volumes. Bone condensation, alveolar ridge, dental arch length discrepancy, ankylosis and root dilaceration are among the local causes of impaction. Dentoalveolar or oral maxillofacial traumas are among the possible local causes of impaction, in variable combinations involving factors such as the kind of trauma or the age of the patient at diagnosis [10].

Surgical intervention for impacted canines can be classified as: exposure for spontaneous eruption, exposure for orthodontic traction with bonding devices and extraction [11-16]. Planning the adequate surgical strategy depends on radiographic analyses or computed tomography (CT), that show the position of the impacted tooth, its eruption path, the stage of root formation, root anatomy [17] and indicate the most adequate orthodontic treatment.

Based on an investigation of the root apex's location and its relationship with the Ennis inverted Y, Puricelli described in 1987 an ortho-surgical procedure to treat upper canine impaction [17]. The eruption of impacted teeth is induced by the guided fracture of the root apex, called apicotomy, resulting from the impact of hammer and chisel, followed by elastic traction of the corono-radicular structure with orthodontic methods [10,17,18]. The present work aims to describe in greater detail the method, that has been succesfully used for the past 20 years.

## Surgical technique

**Figure 1:**
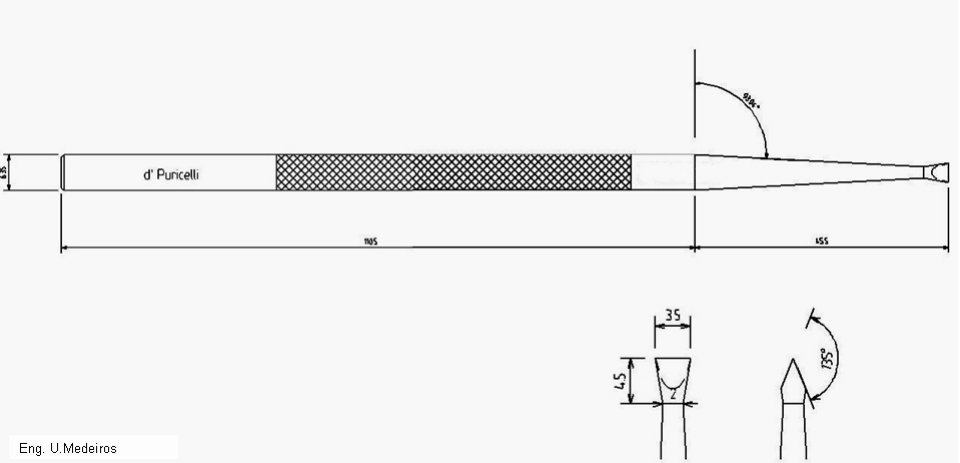
A 16.5 mm long, double-bezel chisel with 135° angle. Its active region is 3.5 mm wide, 4.0 mm thick and 5.0 mm long.

For the surgical fracture of the root apex, the author designed a 16.5 mm long, double-bezel chisel with an angle of 135°. Its active region is 3.5 mm wide, 4.0 mm thick and 5.0 mm long (Figure 1). This design allows a shallow intrusion, with immediate segmentation of the root stucture and minimal risk of damage of the pulp tissue. A surgical hammer, weighing 150 g, is part of the basic instruments involved in the surgical technique.

**Figure 2:**
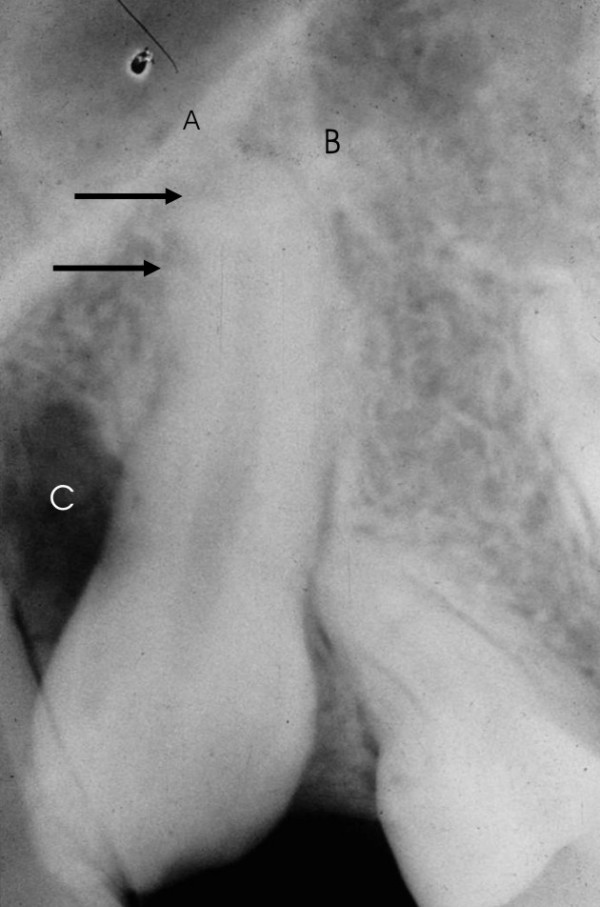
Periapical radiograph for confirmation the presence of the impacted tooth. **aA. **Nasal cortical; **B**. Sinusal cortical (both corticals compose the Ennis inverted Y), the arrow points to dilaceration with possible apical ankylosis; **C**. Bone loss with fibrous scar, possibly due to two previous surgeries.

**Figure 3:**
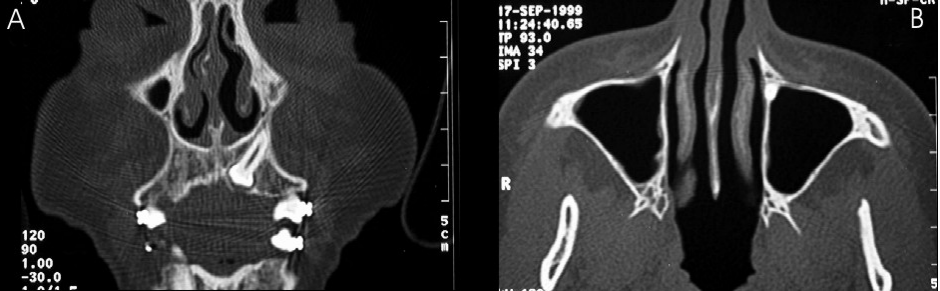
Computed tomography (CT) of the maxilla in coronal (**A**) and axial (**B**) planes allows the visual analysis of the nasal, sinusal and vestibular intercortical relationships. Dilacerations or ankylosis on the root apical region of the impacted canine can be seen (**A**). In both images the absence of spongeous medullary bone around the apical region may be observed.

As part of the surgical planning, clinical examination includes local inspection to confirm the existence of space in the dental arch and orthodontic treatment devices. Panoramic and lateral cephalometric extra-oral radiographs, associated with maxillary occlusal and periapical intra-oral radiographs (Figure 2) are the diagnostic image exams indicated. Computed tomography of the maxilla in coronal and axial planes allows the visual examination of the relationships among nasal, sinusal and vestibular intercorticals. Dilacerations or ankylosis of the apical region of the root of the impacted tooth can be thoroughly observed with these images (Figure 3). The root, contained in a reduced spongeous medullary space, might be connected to one, two or even the three mentioned corticals. Surgery can be performed with local or general anesthesia.

### Incisions

**Figure 4:**
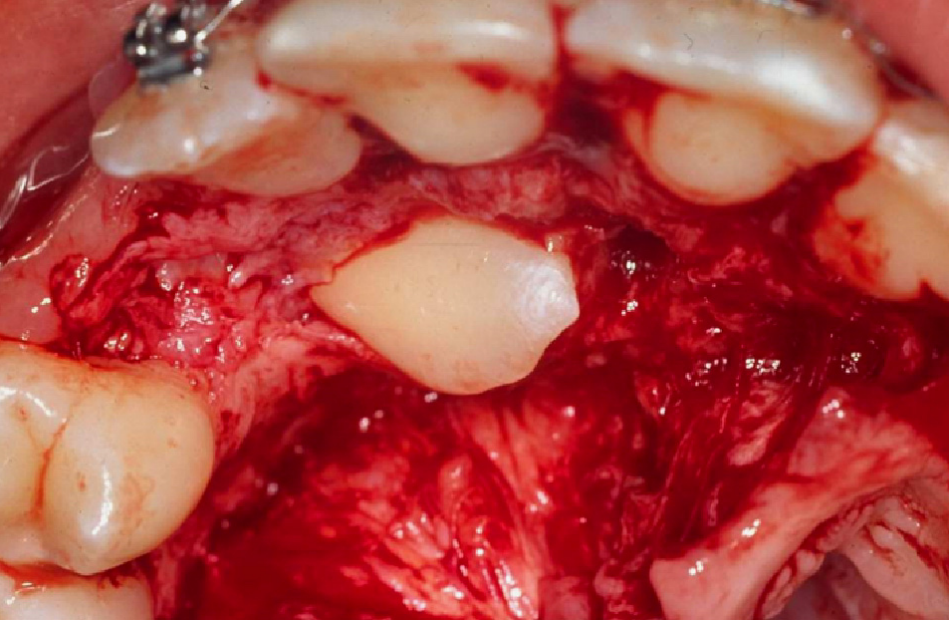
The mucoperiosteal palatine flap should be amply elevated for adequate access, allowing for the visual inspection and technical surgical management of the dental crown.

The incision is planned according to the position of the impacted tooth's crown, as well as to any possible sequelae from previous surgeries. The crown of palatine impacted upper canines or transalveolar situations, with a palatine inclined crown, may be reached by an incision to the palatine gingival papillae. The incision should extend through the space corresponding to two teeth in the mesial and distal directions from the surgical focus (Figure 4). It might be increased if necessary, since it does not present accessory diverticles on its borders.

A linear or semicircular vestibular Partsch's incision [19], modified at the mucogingival level of the ridge, allows the exposure of the apical region of the canine. Limits should include, to the mesial, the pyriform nasal aperture, and to the distal, the apical region of the second premolar or first molar teeth. Linear and palatine incisions are sometimes associated. Neumann's vestibular incision is indicated for teeth with more vertical paths of eruption, since it allows for surgical access to the impacted dental crown and the apical region where the surgical fracture will be performed.

### Exposure of the crown of the impacted tooth

The mucoperiosteal palatine flap should be amply elevated for adequate access and visual inspection of the dental crown (Figure 4). Tissues resulting from the coronary follicle, or scars derived from previous surgeries, should be curettaged, removed and sent for histological examination.

### Surgical fracture of the root apex

**Figure 5:**
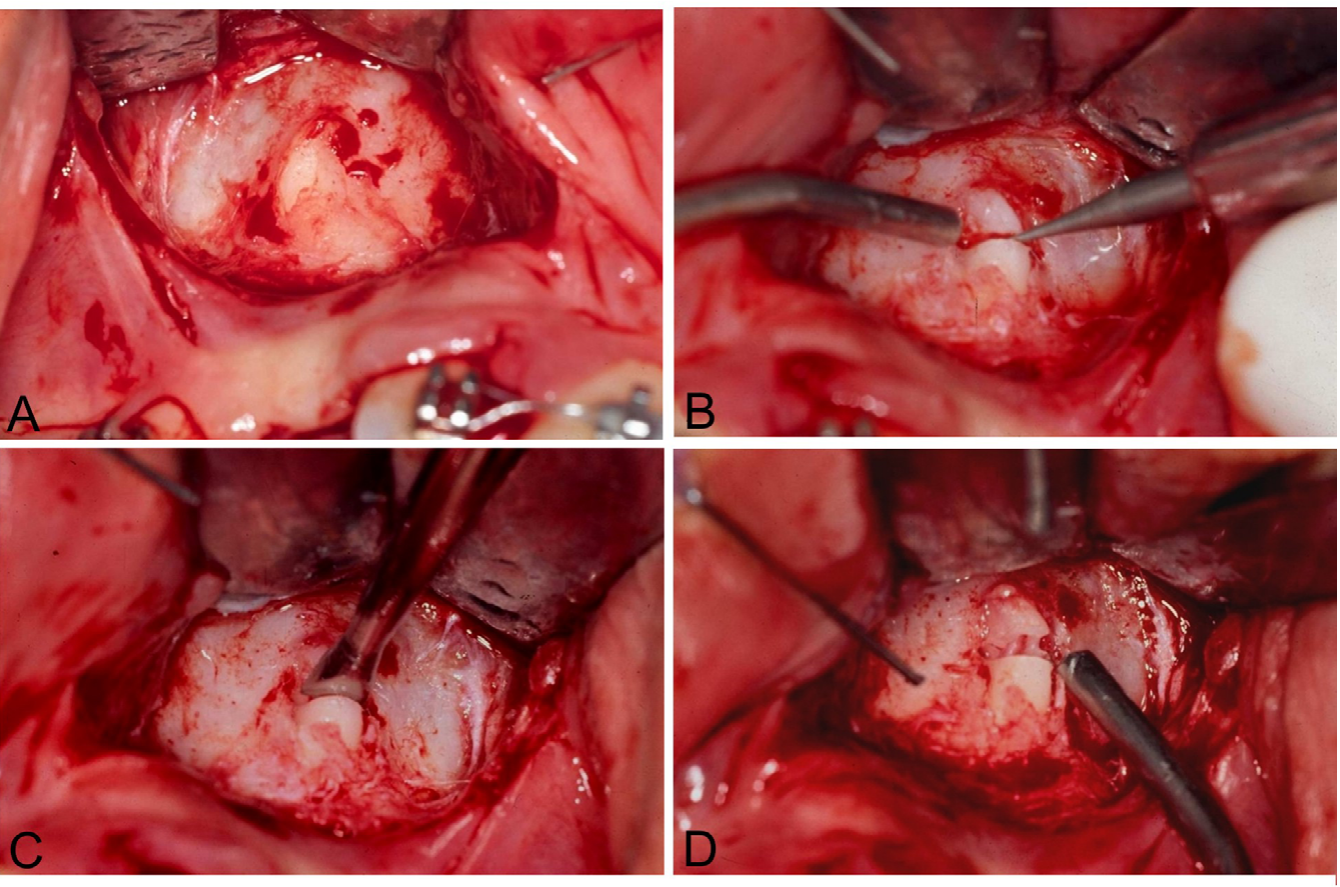
**A. **The bone cortical is removed with a curette under manual pressure, exponsing the apical region; **B. **With a slow rotating, number 1/2 spherical bur and abundant irrigation near the limit between the medial and apical thirds, a transversal groove around 1.0 mm deep is prepared following the limit between the medial and apical third; **C. **The fracture should be produced with the double-bezel chisel; **D**. After the fracture is completed, the separation of the apical root segment becomes clearly visible.

Elevation of the flap, in semilunar shape, or resulting from a Neumann's incision, may be extended to the pyriform nasal aperture, allowing the examination of the bone anatomy in the dental apex area. This region, tridimentionally corresponding to the convergence of the nasal, sinusal and anterior maxillary corticals, contains the apex of the impacted canine, which is included in an area with limited amount of spongeous bone marrow. The determination of the corono-radicular long axis of the first pre-molar allows the identification of the location of the root apex region of the impacted canine. The thin bone cortical is removed under manual pressure, with a curette, exposing the apical region of the canine (Figure 5A). If the root is more deeply located, in the case of a more vertical position of the tooth, osteotomy should be performed with a delicate spherical bur.

The visual identification of a region of brownish colour and a smooth surface inside the bone indicates the location of the root process. The apical region is progressively exposed, in the latero-lateral or mesio-distal and cervical direction, following the inclination of the long axis of the impacted canine's root. Progression in the apical direction should be avoided, due to the risk of damaging the pulp tissues exposed in this region. The limit between the medial third and apical third of the root is identified by a change in colour of the root cement, since the apical region is darker.

With a slow rotating, number 1/2 spherical bur and abundant irrigation near the limit between the medial and apical thirds, a transversal groove around 1.0 mm deep is prepared following the limit between the medial and apical third (Figure 5B). The groove may be up to 1.0 mm deep in the lateral borders, to guide the direction of the fracture and allow adequate segmentation of the dental tissues.

The fracture should be produced with the double-bezel chisel, preferably with a light hit (Figure 5C). If that fails, the groove may be deepened in 0.5 mm, with care, to avoid damage to the pulpar conduct. After the fracture is completed, the separation of the apical root segment becomes clearly visible (Figure 5D). A mild luxation, induced by applying a straight elevator to the tooth crown, can confirm the detachment, since the movement of the corono-radicular process is not reproduced by the apical fractured segment. The insertion of an exploratory probe in the fractured line may also reveal a slight expansion of the groove and separation of the fragments. A transoperative periapical radiography can provide, if necessary, conclusive proof of the resulting fracture. A device for orthodontic traction is then attached to the tooth crown. After irrigation of the surgical wound, the flaps are repositioned and sutured.

### Orthodontic traction

**Figure 6:**
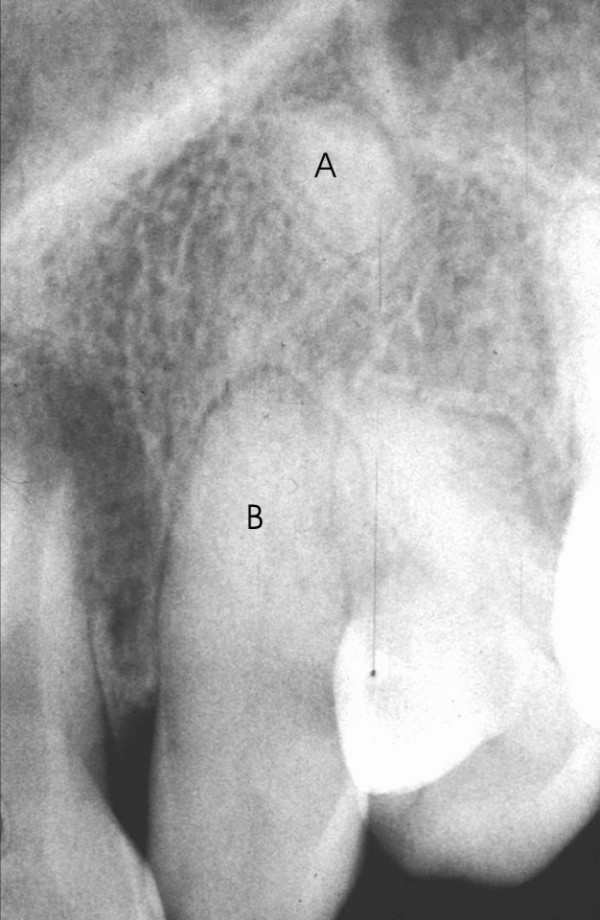
Periapical radiograph after the complete exposure of the tooth crow in the oral cavity and position in the dental arch. **A**. The apical fragment remains close to its preoperative position; **B**. The pulp root canal is almost completely obliterated.

**Figure 7:**
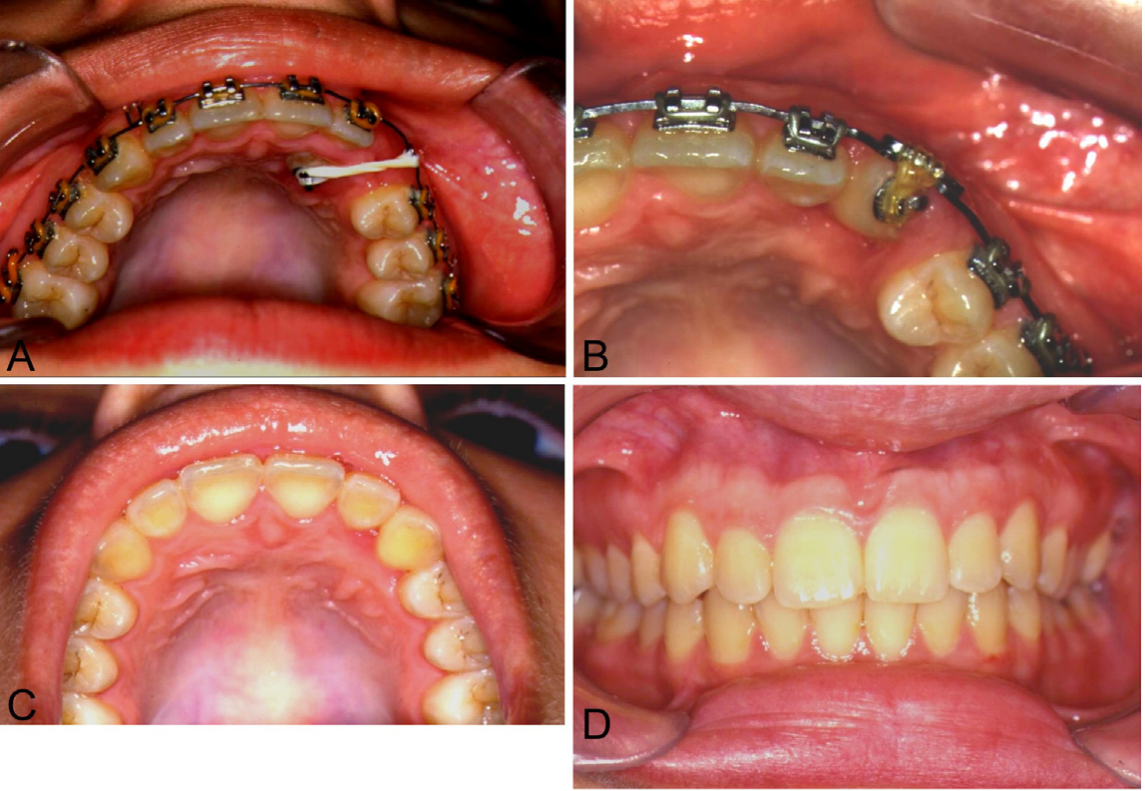
**A**. Clinical aspect of elastic traction; **B**. Tooth in position, included in the dental arch; **C. **Palatine view. Orthodontical treatment concluded. Complete arch without gingival involvement; **D. **View of the occlusion showing excellent gingival borders, without retraction.

Elastic traction should be applied between the fifth and the seventh days after surgery, with an initial strength of 100 to 150 g. During the first 60 days, it should have a more vertical direction, supported by the fixed mandibular orthodontic treatment device. When radiographic examination shows evolution of the case, the strength may be increased or decreased according to individual responses. After exposure of the crown's first two thirds, the use of intermittent force may favour specific orthodontic procedures on the dental arch. Radiologic control examinations are recommended on days 45 and 90 after surgery and then with the appropriate periodicity, of around 120, 180 and 240 days for instance [10,17,18]. The average period for total coronary eruption is eighteen months. Procedures extending beyond this time frame should be associated to orthodontic treatment objectives (Figures 6 and 7).

### Our experience

Table 1 presents a summary of 30 cases submitted to the surgical procedure in the period between 1983 and 2003. The follow-up included clinical and radiographic evaluation. The mean time for complete eruption of the tooth was 17.6 months, and extractions were indicated in 10% of the cases. Except for patient EF, who abandoned the treatment before the minimum time required for tooth eruption had elapsed, all other cases did not need reoperation after apicotomy or endodontic treatment. In our experience, the indications for extraction after apicotomy, in the absence of clinical and radiographic evidences of eruption, occur in low frequency. Unsuccessful cases were almost always due to the lack of commitment by the patient to the mean period of time needed for the process of eruption.

**Table 1:** Distribution of cases of impacted upper canines submitted to apicotomy during the period between 1983 and 2003.

Patient	Age (years)	Sex^1^	Tooth	Previous surgeries Erupt freely (EF) Orthodontic traction (OT)	Time for eruption (months)	Extraction after apicotomy^2^
MF	18	F	23	2xEF ; 1xOT	18	N
IRP	20	F	13	1xEF ; 1xOT	18	N
JAT	19	M	13	2xEF ; 1xOT	18	N
NAB	18	M	23	1xOT	20	N
MSA	17	F	23	2xEF ; 1xOT	16	N
PD	22	M	13	2xEF ; 1xOT	20	N
ALR	18	F	13	2xOT	16	N
AP	17	F	13	2xOT	18	N
TZ	16	F	23	1xEF ; 2xOT	16	N
MRH	27	F	13	1xEF	22	N
NCF	45	F	13	1xEF	18	N
LCV	29	F	23	-	18	N
HA	16	M	23	-	18	N
EF	15	M	23	-	4	Y
PG	34	F	23	-	20	N
FAM	15	M	23	-	19	N
JLF	44	M	13	2xEF ; 2xOT	6	Y
LA	19	M	13	-	18	N
RSS	19	M	13	-	18	N
BAS	23	F	23	1xEF	20	N
MS	23	F	23	1xOT	19	N
SFS	17	F	13	-	18	N
TTK	19	M	13	-	18	N
PK	16	M	23	-	16	N
AA	28	F	13	2xOT	20	N
EM	18	M	13	-	18	N
EM	18	M	23	-	19	N
RP	18	M	13	-	18	N
HS	17	M	23	-	14	Y

^1^M = male, F = female; ^2^Y = yes, N = no.

## Discussion

During the deciduos dentition period, the germ of the permanent upper canine is located in a medullary space of pyramidal, triangular shape, which is delimited by the nasal, sinusal and maxillary anterior corticals. Since developing roots are plastic and shaped, apparently, according to their environment, root apices of upper canines may present dilacerations or ankylosis [17], determined by the close relationship with the above mentioned corticals. Seiler and Pajarola [20] recommend that, in some cases of partial ankylosis, induction of a mild luxation of the tooth before attaching an orthodontic traction device may be useful to induce an adequate eruption. In our experience, the ankylosis process affecting the apical region of the root may, also, be particularly related to luxations performed in previous surgical interventions. Presently, CT analysis and anamnesis with history of previous interventions allow, preoperatively, to presume the diagnosis of apical root ankylosis near the so-called Ennis inverted Y. Routine radiographic examination is less favourable for the observation of this apical ankylosis, but allows the identification of apical dilacerations. The intrabuccal lateral maxillary oclusal incidence is particularly indicated.

The technique of apicotomy proposed by Puricelli in 1987 [17] includes the separation and isolation of the affected apical region, allowing the usage, in the dental arch, of the two root thirds and the intact crown of the upper canine. Application of orthodontic traction five to seven days after the surgical procedure is recommended. According to Andreasen [21], a fractured root region has extensive communication with the periodontal tissues, so that blood supply to the pulp tissue can be more easily reestablished through the periodontal ligament. The development of a pulp tissue edema, which represents another important factor, is resolved by extravasation of fluids through the fracture zone, lowering the pressure on the delicate pulp tissue vessels. Since the vascular circulation is not interrupted in the pulp apical region, necrosis of this segment is very rare in root fractures [22]. The apical fragment does not present infectious postoperative complications [18]. Vitality tests are not indicated in the corono-radicular complex during the traction period, and should be initiated only after complete exposure of the tooth crown in the oral cavity. Negative responses to sensitivity stimuli, however, do not necessarily indicate pulp necrosis [18]. Electric testing of the pulp aims to stimulate an answer from the sensorial fibres in its interior through electrical excitation. The response given by the patient does not suggest pulp integrity, but just the continued existence of vital sensorial fibres. The test does not provide any information about the blood supply available to the pulp, which is the real determinant of pulp tissues vitality. Thermical tests are thus indispensable, and absence of response may indicate a non-vital pulp. The absence of response might also, nevertheless, represent a false-positive result due to excessive calcification, or to an incompletely formed apex, a recent trauma or to the previous use of drugs [22,23]. No pulp alterations, of infectious nature or related to tooth mobility, were observed since the technique was developed. A slight colour modification of the crown, clinically similar to the signs observed in teeth submitted to dental trauma, is seen.

In apicotomy, according to the technique originally described by Puricelli [17], the transversal groove which runs along the root path of the dental element should be shallow over the surgical exposed apical region, so that the pulp structure is not affected. Technically, rupture of the pulp tissue is avoided during the induction of fracture and the light movements aiming at inducing luxation in the fracture line. *In vitro *experiments confirm that the pulp tissue is maintained. The design of the double-bezel chisel proposed by Puricelli is essential for maintenance of integrity of the pulp tissues.

Radiologic analyses show that the corono-radicular and apical fragments may, or may not, undergo increasing separation during the progression of the orthodontic traction. This means that the apical fragments, even if fractured, may be displaced with the corono-radicular portion by the orthodontic traction. A progressive obliteration of the pulp chamber and the root conduct in the corono-radicular element is observed [17,18] (Figures 1 and 6). The phenomenon is well recognized in vital teeth where root fractures were performed [21,22]. In these cases, the conduct may be partially or completely obliterated. Partial obliteration is more frequent in the fracture area and in the apical fragment, whereas total obliteration is characterized by an uniform reduction on the size of the pulp cavity as a whole [21]. The apical region and the periodontal space of the apicotomized erupted tooth present, at radiographic examination, a hard periodontal layer with regular borders [18].

Since 1987, when the technique began to be used, the teeth aligned and leveled in the dental arch have kept stable and functional.

## Conclusion

Apicotomy is a technique which has been successfully used during the past twenty years, for conservative intervention in cases of impacted upper canines with dilaceration or apical root-ankylosis. Currently, it could also be indicated for lower canines. The technique aims at freeing the tooth from its dilacerated or ankylosed portion inducing, thus, its traction and eruption. It was initially indicated after failure of conservative techniques for inducing spontaneous eruption and orthodontic traction. At the moment, image examinations allow precise diagnosis and its indication as a first surgical therapeutic option. The technique is counter-indicated for young patients with incomplete rhizogenesis or for teeth with total root ankylosis.

## Competing interests

The author(s) declare that they have no competing interests.
